# Surgical outcomes of open hip reduction with synovectomy for developmental dysplasia of the hip patient with Juvenile idiopathic arthritis: A case report

**DOI:** 10.1016/j.ijscr.2020.06.041

**Published:** 2020-06-17

**Authors:** Thamer S. Alhussainan, Abdullah M. Alghamdi, Rakan A. Almogbel

**Affiliations:** a Department of Orthopedic Surgery, Pediatric Orthopedic Unit, King Faisal Specialist Hospital and Research Center, Riyadh, Saudi Arabia; b Department of Surgery, Division of Orthopedic Surgery, King Abdulaziz Medical City, Ministry of National Guard Health Affairs, Riyadh, Saudi Arabia

**Keywords:** Developmental dysplasia of the hip, Hip dysplasia, Juvenile idiopathic arthritis, Hip synovectomy, Case report

## Abstract

**Introduction:**

Developmental dysplasia of the hip (DDH) is a common hip disorder and its association with other musculoskeletal, genetic, and neurological diseases were well described in the literature. Juvenile idiopathic arthritis (JIA) is a rare rheumatological condition, and its presence in a Developmental dysplasia of the hip (DDH) child makes this a very challenging case.

**Presentation of the case:**

This case report is describing the presentation of 9 months old girl to orthopedic service referred from the pediatric rheumatology clinic after diagnosing her and starting the treatment for Juvenile idiopathic arthritis (JIA). Bilateral neglected Developmental dysplasia of the hip (DDH) - International Hip Dysplasia Institute (IHDI) type 4- was detected during her clinical and radiological assessment, necessitating surgical management after controlling her Juvenile idiopathic arthritis (JIA). The surgical procedure and its clinical and radiological outcomes more than four years after her surgical treatment are described in detail in this report as well.

**Discussion:**

The management of Developmental dysplasia of the hip (DDH) associated with Juvenile idiopathic arthritis (JIA) has no established guidelines in literature. Here, we share our experience in managing such rare cases. We believe that medical control of Juvenile idiopathic arthritis (JIA) before proceeding for open reduction of Developmental dysplasia of the hip (DDH) is the key to successful results.

**Conclusion:**

The reported case is uniquely having both Developmental dysplasia of the hip (DDH) and Juvenile idiopathic arthritis (JIA) treated with open reduction, pelvic osteotomy, and femoral shortening resulting in outstanding clinical and radiological outcomes.

## Highlights

• Most of the characters is used to write the diagnosis name (developmental dysplasia of the hip + juvenile idiopathic athritis).
• DDH association with JIA is very rarely reported in the literature. Here we describe our experience and results.
• Management of DDH associated with JIA has no established guidelines in literature. Here, we share our experience in managing such rare cases.
• We believe that medical control of JIA before proceeding for an open reduction of DDH is the key to successful results.

## 1 Introduction

Developmental dysplasia of the hip (DDH) is a common pediatric orthopedic disorder involving a spectrum of the hip anomaly with variant severity from simple acetabular dysplasia to fixed irreducible hip dislocation [1] Depending on different factors, the incidence of Developmental dysplasia of the hip (DDH) varies from 1 to 34 per 1000 births worldwide [2]. Juvenile idiopathic arthritis is joint arthritis of unknown etiology starting before 16 years of age and lasting for at least six months [3]. The prevalence varies from 16 to 150 per 100,000 children [4].

Medical treatment had been considered the backbone for managing Juvenile idiopathic arthritis (JIA). Medical treatment of the disease is the key and assists alleviating the symptoms and delaying joint destruction. That includes nonsteroidal anti-inflammatory, systemic corticosteroids, methotrexate, sulfasalazine, and biological treatment [3].

Synovectomy of the hip in patients with juvenile idiopathic arthritis is a well-known treatment for the management of refractory cases of hip synovitis that is not responding to conservative management [4].

One of the main objectives of surgically treating neglected Developmental dysplasia of the hip (DDH) patients is to obtain stable mobile and painless hips, which might not be achieved in a joint involved in Juvenile idiopathic arthritis (JIA).

The purpose of this study is to report an unusual case of a patient well known to have familial juvenile idiopathic polyarticular arthritis and presented with bilateral developmental dysplasia of the hip. The current case report work has been reported in line with the Surgical Case Report Guidelines SCARE 2018 criteria [5].

## 2 Presentation of case

A 7-year-old Saudi girl, diagnosed at the age of nine months with both familial juvenile idiopathic polyarticular arthritis and bilateral developmental dysplasia of the hip (DDH). She is also known to have hypertension, congenital solitary left kidney.

Our colleagues from pediatric rheumatology started treating her medically at the age of nine months initially with Indomethacin, Prednisone, and Alfacalcidol drops.

**Fig. 1:**
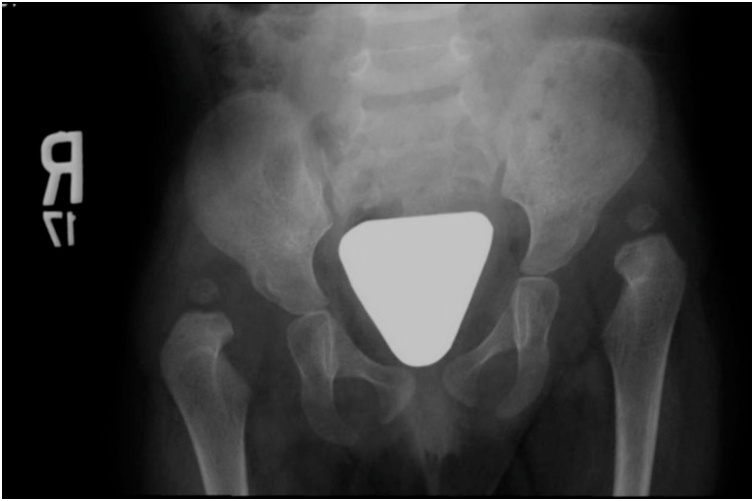
Pelvis AP x-ray showing bilateral hips dislocated DDH at age of 2 years, Right Tonnis 3, left Tonnis 4.

The patient was diagnosed with bilateral developmental dysplasia of the hip (DDH) at the age of 9 months. And as the patient had inflamed hips and knees due to active polyarthritis status, we decided to delay treatment of her bilateral developmental dysplasia of the hip (DDH) until pediatric rheumatologist controlled her disease. And At the age of 16 months, she was still having active arthritis involving both wrists, small joints of both hands as well as bilateral knee and hips joints. She was labeled as a refractory disease with partial response to Non-Steroidal Anti-Inflammatory, systemic steroid, and Methotrexate medications. At that time, she had IHDI grade 4, bilateral dislocated developmental dysplasia of the hip [6] (Fig. 1).

**Fig. 2:**
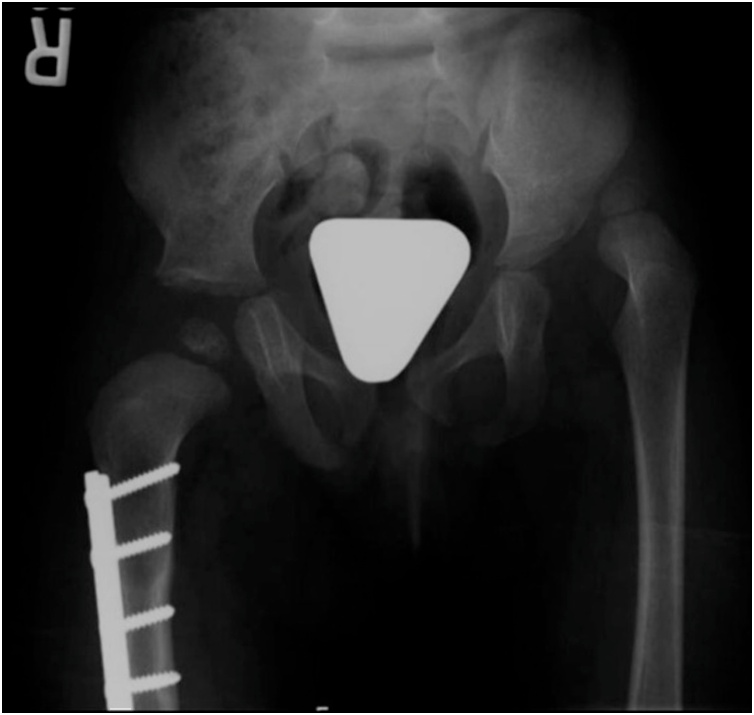
Pelvis AP x-ray at age of 2 ½ years after Right hip open reduction, adductor tenotomy, pelvis osteotomy, femoral shortening and hip Spica.

**Fig. 3:**
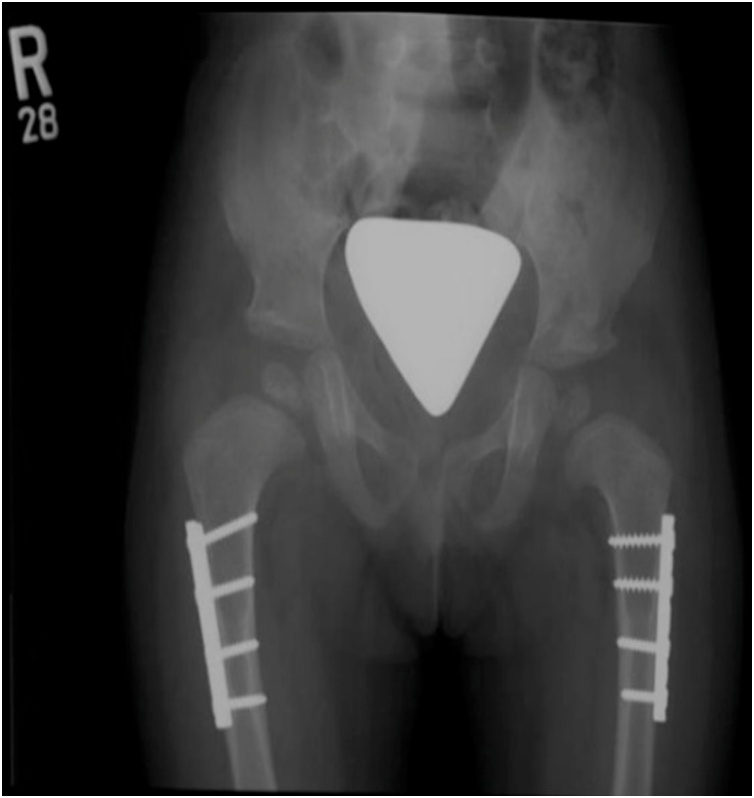
Pelvis AP x-ray at age of 3 years after bilateral hips open reduction, adductor tenotomy, pelvis osteotomy, femoral shortening and hip Spica, Right hip 12 months post- operatively, left hip 6 months post-operatively.

Her juvenile idiopathic arthritis was well controlled medically at the age of two with Methotrexate, Abatacept, and antihypertensive medication. At the age of two years old, surgical reduction was indicated and was operated by an experienced surgeon in Developmental dysplasia of the hip (DDH) surgery operating such cases for more than ten years. Initially, she underwent right hip open reduction, adductor tenotomy, and pelvic osteotomy with bone allograft, femoral shortening osteotomy and plate fixation, and spica cast application for 6 weeks then 4 weeks of broomstick cast for 4 weeks utilizing the technique of Wade et al. [7] (Fig. 2). Seven months later, she had the left hip joint similarly reduced (Fig. 3).

The second surgery was postponed 7 months from the first surgery to assure the full recovery of operated hip ROM and allow sufficient period for full weight-bearing walking activity to recover the bone density from the expected disuse osteopenia, which will make the associated bony procedures more efficient and carries lower complications rate.

During the open hip reduction for both hips, we faced an unusual intra-articular obstacles in the form of adhesions and an abundant amount of synovitis immediately after opening the hip joint capsule - which is an integral part of open hip reduction in neglected Developmental dysplasia of the hip (DDH) cases-. Synovectomy was done, and tissue was sent for histopathology study confirming severe synovitis.

Usually, after surgical treatment of neglected Developmental dysplasia of the hip (DDH) patients, the routine referral to physiotherapy services is not carried out especially if the child is living far from the hospital and most of the children treated for Developmental dysplasia of the hip (DDH) alone had smooth recovery of hip function. In this case, because of the concern about the recovery of hips range of motion (ROM), an early referral to physiotherapy was done immediately after removal of the cast for each side to start gait training, recover ROM, and strengthen the hip and knee muscles.

**Fig. 4:**
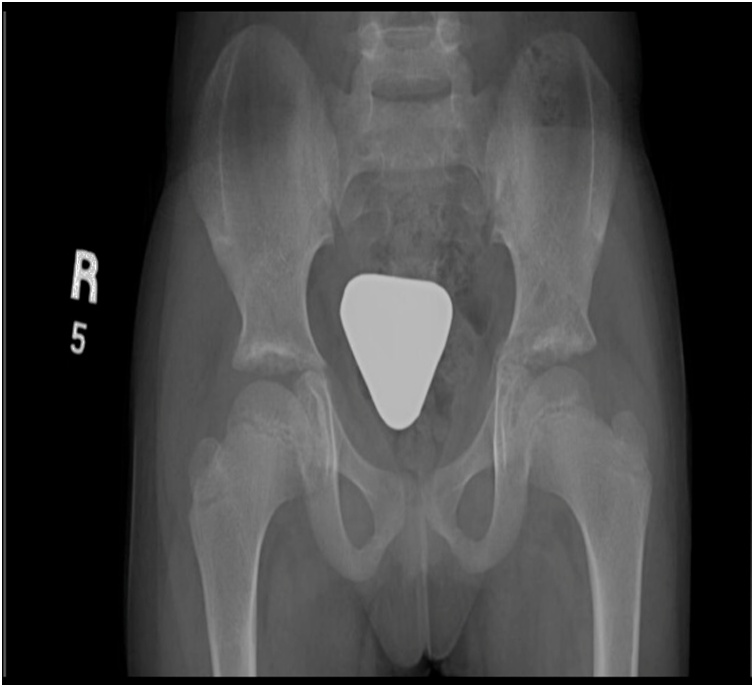
Pelvis AP x-ray at age of 7 years after removal of femur implants.

Two years later, the proximal femurs plates and screws - at the shortening osteotomy site - were surgically removed. The patient remained following in the clinic utill the writing of this report. On her last presentation in the clinic, at the 7th year of her age, she had grown well and completely independent in her daily activities. She is free of any joints pain and deformity, has full range of motion and normal gait (Fig. 4).

Final results were amazingly excellent with non-painful hips, full range of motion, functionally scoring grade A for both hips in the McKay scoring system [8]. Hips x-rays showed covered femoral heads with congruent joints and no arthritic changes or signs, which is graded as Severn Type 1A hip bilaterally [9]. Regarding her Juvenile idiopathic arthritis, she is maintained on Abatacept intravenous monthly injection in addition to antihypertensive treatment.

## 3 Discussion

Juvenile idiopathic arthritis (JIA) is a rheumatologic disorder characterized by autoimmune inflammation of synovial joints [10]. Globally, Juvenile idiopathic arthritis (JIA) is considered the commonest chronic arthritis amongst the pediatric population [3]. Its common features are joint pain, swelling, stiffness, and reduced function caused by inflamed synovial tissue [3].

Collectively, it results in disability and early joint damage [4]. It strikes larger joints, and the knee is being the most affected [11]. The hip joint is affected in 70% of total Juvenile idiopathic arthritis (JIA) subtypes, and 50% of the systemic form [2]. Synovial tissue inflammation is a characteristic feature of Juvenile idiopathic arthritis (JIA) [12].

Medical treatment had been considered the backbone for managing Juvenile idiopathic arthritis (JIA). Control of the disease is the key, and promotes in the alleviation of symptoms and delaying joint destruction. That includes non-steroidal anti-inflammatory, systemic corticosteroids, methotrexate, sulfasalazine, and biological treatment [3].

Open and arthroscopic synovectomy has been performed in refractory cases that are thought to improve symptoms [3]. Hip joint synovectomy had been performed as a mode of treatment in severe and intractable cases aiming for pain reduction, increasing joint range of motion, and improving functional status [4]. Carl et al. showed significant improvement after synovectomy in general, and more specifically, in terms of pain, range of motion, and ambulation. Around 85% exhibited major recovery, 12% had a fair recovery, and 3% were unsuccessful [4].

Developmental dysplasia of the hip (DDH) is a spectrum of hip developmental anomaly from simple acetabular dysplasia to full dislocation, happening in utero or neonatally. The disease involves the developmental interruption of acetabulum and femoral head as well [13].

Treatment of Developmental dysplasia of the hip (DDH) in a walking child is different and more challenging than the earlier stage. Soft tissue becomes more rigid, forming intracapsular and extracapsular obstacles to reduction necessitating surgical open hip reduction [14].

Open hip reduction is the treatment of choice for Developmental dysplasia of the hip (DDH) patients who failed closed reduction or exceeded 18 months of age, with a success rate of maintaining a reduction in 99% of cases, and 12% of variable subluxated joints [15]. Open reduction is usually combined with a pelvic osteotomy to address the associated acetabular dysplasia and sometimes femoral shortening osteotomy that will facilitate the reduction without a significant pressure load over the initially proximally migrated femoral head when they are reduced to the level of the acetabulum. All the previously mentioned procedures are safely done simultaneously [14].

Modified Smith-Peterson anterolateral approach is usually the approach of choice in patients more than 18 months old, and it’s very useful as it allows for a concomitant pelvic osteotomy and capsulorrhaphy [16]. Klisic et al. described femoral shortening osteotomy in late presenting Developmental dysplasia of the hip (DDH) that facilitates reduction and decreases the rate of Avascular Necrosis [17]. Femoral osteotomy should be used whenever a substantial force is needed to reduce the femoral head into the acetabulum.

Pelvic osteotomy should be done for persistent acetabular dysplasia when acetabular coverage is deficient. Recently, there is a tendency to include acetabular procedure at the same time of open reduction to obtain better intra-operative hip stability and maximize the potential for the development of normal acetabulum [18].

Complications after open reduction of Developmental dysplasia of the hip (DDH) include osteonecrosis, re-dislocation, and stiffness. A study by Castaneda et al. discussing complication post-open reduction of late presenting Developmental dysplasia of the hip (DDH) showed 18% risk of residual dysplasia or subluxation, and 5.5% of re-dislocation in patients who had open reduction alone, and 1.9% in patient who had both open reduction and pelvic osteotomy, and both, re-dislocation and subluxation, were associated with increased age at the time of surgery, they also noted that the rate of Avascular Necrosis (AVN) post-open reduction of Developmental dysplasia of the hip (DDH) was 14% [19]. Re-dislocation post-open reduction has been associated mainly with failure to remove all obstacles, including ligamentum teres, transverse acetabular ligament, and pulvinar [16].

The patient we reported was 2 years old when she had her Juvenile idiopathic arthritis (JIA) medically controlled, and soon she underwent successful bilateral hip surgeries with 7 months interval to allow effective recovery. That included open reduction, adductor tenotomy, and pelvic osteotomy with bone allograft, femoral shortening osteotomy, plate fixation, and spica cast application. The severe synovitis she had manifested in joints pain and swelling, and might be a factor in advancing her Developmental dysplasia of the hip (DDH) stage pre- operatively. The thick synovial tissue that was excised intraoperatively, as part of the procedure, may have improved her post-operative hip status and function. The treatment plan went uneventfully with no apparent complication. Instead, she had great outcomes. She is now enrolled in school and independently performing daily activities of her age group.

The results were outstanding clinically and radiologically. We can state that it is not contraindicated to perform Developmental dysplasia of the hip (DDH) open reduction on Juvenile idiopathic arthritis (JIA) diseased patients, and it is safely done in the appropriate time and settings. In our belief, the medical control of Juvenile idiopathic arthritis (JIA) was the key to success in this dilemma, and such exceptional results cannot be reproduced without such successful medical control. We strongly advise the treating surgeon for such a clinical challenge not to rush the surgical treatment until the medical treatment confirms its control on the disease activity. Otherwise, the good outcomes are less likely to be achieved. We also believe that the solid base for optimal management in these cases is the proper communication and coordination between the Pediatric Rheumatologist, Pediatric Orthopedic surgeon, and rehabilitation specialist. We recommend and hope to have future case series and larger sample size studies to obtain a wider view of this unique combination of diseases.

## 4 Conclusion

No previous guidelines, to our knowledge, have highlighted the outcomes of surgical treatment in combined Developmental dysplasia of the hip (DDH) and Juvenile idiopathic arthritis (JIA) cases. The reported case is uniquely having both Developmental dysplasia of the hip (DDH) and Juvenile idiopathic arthritis (JIA) treated with open reduction, pelvic osteotomy and femoral shortening resulting in outstanding clinical and radiological outcomes. The key to successful outcome in our belief and recommendation is to control the medical aspect of Juvenile idiopathic arthritis (JIA) before attempting open reduction of Developmental dysplasia of the hip (DDH).

## Declaration of Competing Interest

None.

## Sources of funding

None.

## Ethical approval

The study was approved by institutional review board by king faisal specialist hospital and research centre.

## Consent

Written informed consent was obtained from the patient’s father for publication of this case report and accompanying images. A copy of the written consent is available for review by the Editor-in-Chief of this journal on request.

## Author contribution

Thamer S. Alhussainan: Surgeon, Supervisor and editor.

Abdullah M. Alghamdi: Reviewed the literature, writer and data collector.

Rakan A. Almogbel: Writer, organizer and paper submitter.

## Registration of research studies

Not required.

## Guarantor

Rakan A Almogbel.

## References

[1] Elizabeth F., Khanna G., J MSA (2013). Imaging of Juvenile idiopathic arthritis: a multimodality. RadioGraphics.

[2] Inui K., Maeno T., Tada M. (2004). Open reduction of the dislocated hip in Juvenile idiopathic arthritis : a case report. Mod. Rheumatol..

[3] Giancane G., Consolaro A., Lanni S. (2016). Juvenile Idiopathic Arthritis : Diagnosis and Treatment.

[4] Carl B.H., Schraml A., Swoboda B., Hohenberger G. (2007). Synovectomy of the hip in patients with Juvenile rheumatoid arthritis. J. Bone Jt. Surg..

[5] Agha R.A., Borrelli M.R., Farwana R., Koshy K., Fowler A., Orgill D.P., For the SCARE Group (2018). The SCARE 2018 statement: updating consensus Surgical CAse REport (SCARE) guidelines. Int. J. Surg..

[6] Narayanan U., Mulpuri K., sankar W.N. (2015). Reliability of a new radiographic classification for developmental dysplasia of the hip. J. Pediatric Orthop..

[7] Wade W.J., Alhussainan T.S., Al zayed Z., Hamdi N., Bubshait D. (2010). Contoured iliac crest allograft interposition for pericapsular acetabuloplasty in developmental dislocation of the hip: technique and short-term results. J. Child. Orthop..

[8] McKay D.W. (1974). A comparison of the innominate and the pericapsular osteotomy in the treatment of congenital dislocation of the hip. Clin. Orthop. Relat. Res..

[9] Severin E. (1941). Contribution to the knowledge of congenital dislocation of the hip joint; late results of closed reduction and arthrographic studies of recent cases. Acta Chir. Stand..

[10] Aguiar F., Brito I. (2016). Structural damage to the hip in systemic juvenile idiopathic arthritis: a case of regression with Anakinra. Reumatol. Clínica.

[11] Al-hemairi M.H., Albokhari S.M., Muzaffer M.A. (2016). The pattern of juvenile idiopathic arthritis in a single tertiary center in Saudi Arabia. Int. J. Inflam. Vol..

[12] Moued M.M., Al-saggaf H.M., Habib H.S. (2013). Oligoarticular juvenile idiopathic arthritis among Saudi children. Ann. Saudi Med..

[13] Swarup I., Penny C.L., Dodwell E.R. (2017). Developmental dysplasia of the hip : an update on diagnosis and management from birth to 6 months. Curr. Opin. Pediatr..

[14] Czubak J., Kowalik K., Kawalec A. (2018). Dega pelvic osteotomy : indications, results and complications. J. Child. Orthop..

[15] Schaeffer E.K., IHDI study group, Mulpuri K. (2018). Developmental dysplasia of the hip: addressing evidence gaps with a multicentre prospective international study. MJA.

[16] Vitale M.G., Skaggs D.L. (2001). Developmental dysplasia of the hip from six months to four years of age. J. Am. Acad. Orthop. Surg..

[17] Klisic P., Jankovic L. (1976). Combined procedure of open reduction and shortening of the femur in treatment of congenital dislocation of the hips in older children. Clin. Orthop. Relat. Res..

[18] Gillingham B.L., Sanchez A.A., Wenger D.R. (1999). Pelvic osteotomies for the treatment of hip dysplasia in children and young adults. J. Am. Acad. Orthop. Surg..

[19] Castañeda P., Masrouha K.Z., Vidal Ruiz C., Moscona-Mishy L. (2018). Outcomes following open reduction for late-presenting developmental dysplasia of the hip. J. Child. Orthop..

